# The Influence of Eyelid Position and Environmental Conditions on the Corneal Changes in Early Postmortem Interval: A Prospective, Multicentric OCT Study

**DOI:** 10.3390/diagnostics12092169

**Published:** 2022-09-07

**Authors:** Matteo Nioi, Pietro Emanuele Napoli, Roberto Demontis, Alberto Chighine, Fabio De-Giorgio, Simone Grassi, Vincenzo Scorcia, Maurizio Fossarello, Ernesto d’Aloja

**Affiliations:** 1 Forensic Medicine Unit, Department of Clinical Sciences and Public Health, University of Cagliari, 09124 Cagliari, Italy; 2 Eye Clinic, Department of Surgical Science, University of Cagliari, 09124 Cagliari, Italy; 3 Legal Medicine, Department of Health Surveillance and Bioethics, Università Cattolica del Sacro Cuore, Fondazione Policlinico Universitario Agostino Gemelli IRCCS, 00168 Rome, Italy; 4 Section of Forensic Medical Sciences, Department of Health Sciences, University of Florence, 50121 Florence, Italy; 5 Department of Ophthalmology, University ‘Magna Græcia’ of Catanzaro, Viale Europa, 88100 Catanzaro, Italy

**Keywords:** postmortem ocular changes, postmortem optical coherence tomography, forensic pathology, cornea, postmortem eye, forensic imaging, postmortem interval, corneal transplant

## Abstract

In the current study, using portable optical coherence tomography, we evaluated 46 corneas of 23 individuals in a multicenter setting during the first 17 h after death. Twenty-three eyes were kept open, and twenty three were kept closed. Furthermore, the experiment was carried out for 12 samples in summer and 11 in winter. Our data show that postmortem corneal alterations largely depend on the phenomena of dehydration (in particular in open eyes) and swelling of the stroma in closed eyes, probably due in the first phase to hypoxia/anoxia and subsequently to the passage by osmosis of the aqueous humor from the anterior chamber to the corneal tissue. Our findings could have significant repercussions in forensic pathology for estimating the postmortem interval and transplantation to optimize the conservation of the tissue before the explant.

## 1. Introduction

The macroscopic phenomenon of corneal opacification in the postmortem period is described abundantly in the literature and is relevant to every professional dealing with forensic pathology and corneal transplants. However, few studies have analyzed the genesis of this phenomenon in depth. This is due to various reasons, such as the disfigurement caused by postmortal enucleation and the need for uncommon levels of expertise to perform histological or immunohistochemical analyses on corneal tissue.

Consequently, forensic research on the eye in postmortem intervals focused almost exclusively on the biochemical alterations affecting the aqueous and vitreous [1,2,3,4,5,6,7,8,9].

In recent decades, diagnostic technologies based on optical coherence tomography (OCT) imaging have become indispensable in ophthalmology, studying the retina and the anterior segment [10,11,12].

Only in recent years, with a great delay compared to the clinical application, has the use of this technology arrived in forensic medicine (at least in forensic medicine research) [13].

In particular, the feasibility and reliability of this new device in the study of the cornea in situ after death, both using animal models and human samples, were examined by performing various experiments [14,15]. Some studies evaluated cornea morphology using animal samples or a limited number of humans [16,17,18,19].

The current work aimed to describe the morphological alterations of the cornea that can be evaluated in the first 17 h after death by portable OCT and to evaluate whether these data could have applications in forensic pathology and/or corneal transplantation.

## 2. Materials and Methods

**Figure 1:**
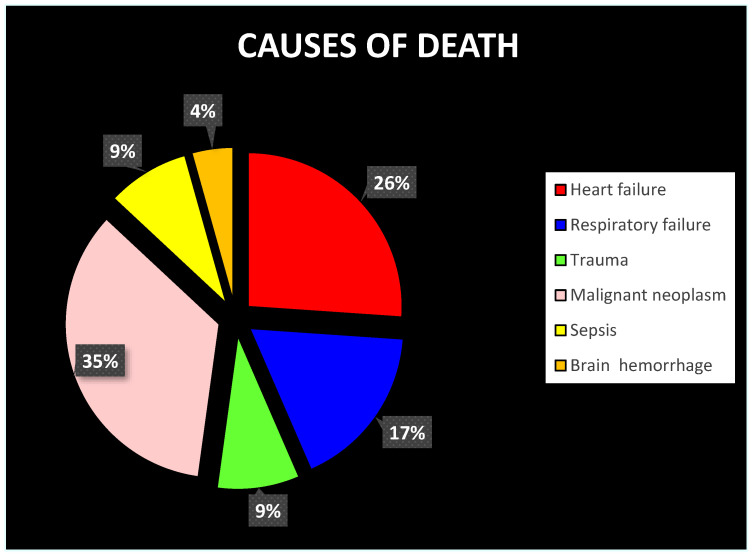
Causes of death among the sample.

A total of 23 bodies (14 males, 9 females) were examined once they arrived at the morgue of two university teaching hospitals (“Fondazione Policlinico Gemelli”, Catholic University of Rome, and the “Policlinico di Monserrato”, University of Cagliari) in two different periods between 1 and 30 July 2017 and between 8 and 31 January 2018. The average age was 71.26 ± 14.29 years. The causes of death are summarized in Figure 1.

After death, bodies were transferred to a room with a known humidity and temperature and monitored over time. The range of temperatures was 20–23 °C (22.12 ± 0.64 °C) in July and 15.4–16.9 °C (16.09 ± 0.68 °C) in January. The range of humidity was 36–56% (47.45 ± 6.23%) in July and 45–60% (49.83 ± 3.71%) in January. In all cases, the postmortem interval and clinical data were known. In no case were eye drops used to avoid altering the local state of the corneal tissue. Overall, 32 eyes (16 corpses) were studied in Rome, and 14 eyes (seven corpses) were studied in Cagliari.

Only samples with a history irrelevant to systemic pathologies with ophthalmological repercussions were considered. Therefore, subjects with external ocular diseases, any evidence of lid abnormality, history of corneal surgery, or previous topical or systemic medication were excluded in both centers. All the corpses that arrived in the morgue after the third hour since death were also excluded which is the time-point in which, based on studies with an animal model, it was shown that endothelial decompensation was already in progress).

In all corpses, the right eye was closed using suitable supports, and the left was kept open. From the arrival until the restitution of the body, corneal imaging was performed using a portable spectral-domain OCT (SD-OCT) device (iVue SD-OCT, Optovue Inc, Fremont, CA) every hour in the first 17 h postmortem to evaluate the impact of the eyelid opening and environmental conditions on the occurrence of tissue changes.

Starting from animal model data available in the literature, the interval from time 0 to 3 h was considered as the baseline. Four other intervals were then considered: from the 3rd to the 6th hour, from the 6th to the 9th hour, from the 9th to the 12th hour, and from the 12th to the 17th hour. The data thus obtained were processed and interpreted by three OCT ophthalmologists and six forensic pathologists.

## 3. Results

The results are described in Table 1 and Table 2.

### 3.1. Baseline

**Figure 2:**
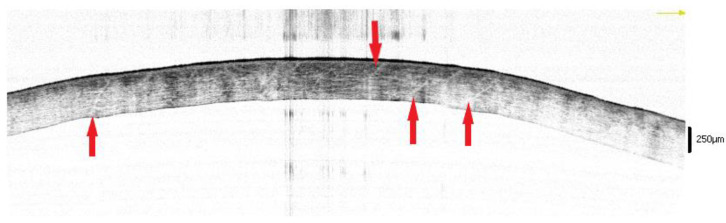
The black and white image highlights the presence of X- and V-shape and linear stromal striae (indicated with red arrows). This sign, initially considered an imaging artifact, constitutes a structural feature of the corneal tissue that has only recently been discovered (and it is still under study).

At baseline, all corneas had the following characteristics:

(a) Persistence of the tear film.
(b) Preservation of the epithelial state.
(c) Homogeneity and preservation of the physiological structure of the corneal stroma. The presence of alphabetic-shaped stromal striae (SS) was osservable in a variable (Figure 2) number of cases.
(d) Morphological integrity of the endothelium.

### 3.2. Open Eyes

#### 3.2.1. The 3–6 h Interval

**Figure 3:**
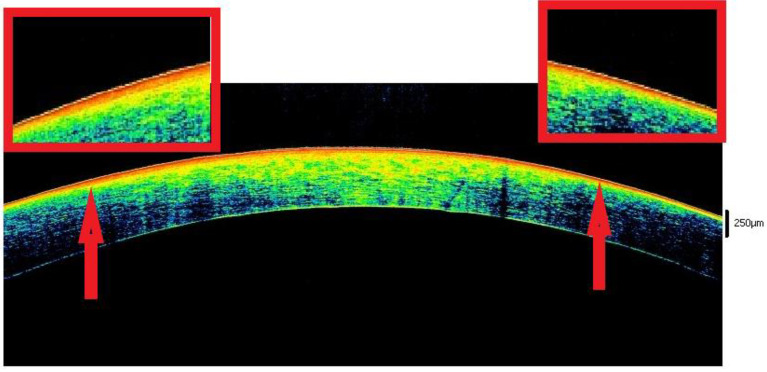
Binary sign. In open eyes, the phenomena of dehydration lead to the drying of the tear film (outer line of hyper-reflectivity). At the same time the appearance of hyper-reflectivity in the anterior surface of the cornea can be observed (inner line of hyper-reflectivity). These phenomena favor the formation of the typical “binary” pattern (indicated by red arrows) that characterizes all corneas exposed to the environment in the interval between the third and sixth hour.

With open eyes, there was an overall advancement in the reflectivity of the epithelium in all cases. Up to the 6th hour, the epithelium (a hypo-reflective layer) was detected within a binary pattern, i.e., two hyper-reflectivity lines corresponding to the tear film (outer) and a basal membrane state (inner) in 100% of cases (23/23) (Figure 3) [20].

In all cases, there was a thinning of the stroma over time. From the morphological point of view, in the context of the stromal tissue, a differentiation was observed between anterior stroma (hyper-reflective) and posterior stroma (hypo-reflective). In 78.2% of cases (18/23), the border between anterior and posterior stroma morphology showed a sawtooth appearance. Within the tissue, in 52.1% (12/23) of cases, the presence of linear, X-shaped, or V-shaped SS was detected. The endothelium in all cases became hyper-reflective with the presence of hyperreflective spots.

#### 3.2.2. The 6–9 h Interval

In the interval from the 6th to the 9th hour, at the epithelial level, there was the disappearance of the previously described binary pattern. In all cases, the morphology of the epithelium was characterized by a single hyper-reflective layer. A further decrease in stromal thickness was recorded in association with the extension of the anterior hyper-reflectivity area. In 52.1% of the cases, the presence of SS was observed. However, the morphological characteristics of the endothelium remain unchanged.

#### 3.2.3. The 9–12 h Interval

**Figure 4:**
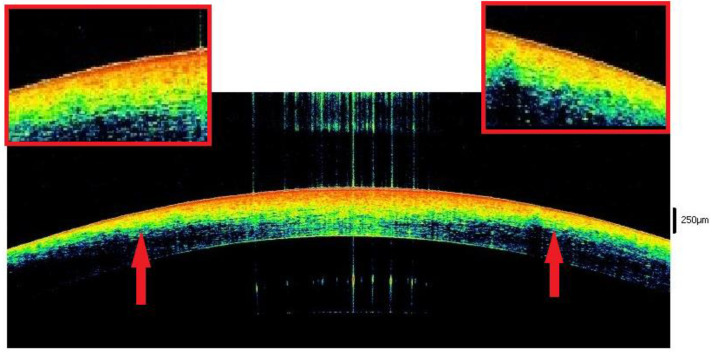
This image shows the progress of the dehydration phenomenon which also accentuates the physiological structural differences between the anterior and posterior stroma in opened eyes. The image shows the presence of an anterior hyper-reflective stroma which was separated from a posterior hyporeflective area by an irregular line with a saw-tooth morphology (indicated by red arrows).

No significant changes in the epithelium were observed from the 9th to the 12th hour. The hyper-reflective part of the stroma was enlarged, thus touching the endothelium in all cases. The presence of linear SS was found in 100% of cases. In the evaluation of the paracentral stromal zone, an area of hyper-reflectivity was observable, with a greater extension in the anterior part of the stroma, near the ocular surface. Overall, the hyper-reflectivity area formed a trapezoid geometry that was bat-like shaped, with scalloped and blurred lateral borders, i.e., sawtooth morphology, in 78.2% of cases (18/23) (Figure 4). Starting from the 9th hour, a macroscopic alteration of the corneal curvature was observed in about 21.7% (5/23) of cases.

#### 3.2.4. The 12–17 h Interval

There were no major changes in the epithelium from the 12th to the 17th hour. Conversely, the formation of a hypo-reflective posterior zone in the stroma was gradually observed in 100% of cases. The SS and the sawtooth morphology in the peripheral parts were detected in all examined samples. The presence of a hypo-reflective stromal contour highlighted the hyper-reflectivity of the endothelium. The regularity of the corneal curvature was lost in eight of twenty three (34.7%) cases.

The main changes affecting the open eyes are summarized in Table 1.

### 3.3. Closed Eyes

#### 3.3.1. The 3–6 h Interval

**Figure 5:**
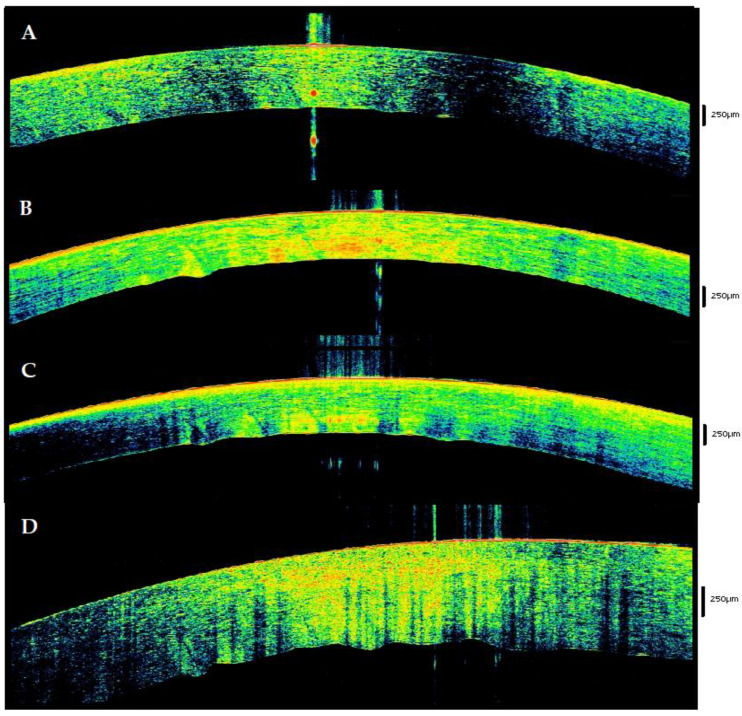
Nioi–Napoli sign. The Nioi–Napoli sign is a morphological feature occurring in the posterior part of the cornea in the postmortem period. This sign is likely due to two different phenomena that take place in distant portions of the cornea: the dehydration of the anterior tissues and the edema of the posterior part (which has repercussions in all contiguous tissue of the stroma, epithelium included. From the morphological point of view, it consists of different phases or stages: (**A**) “Stade I”: initial posterior waving. (**B**) “Stade II” posterior waving with 1 peak. (**C**) “Stade III” posterior waving with multiple peaks and initial stromal swelling. (**D**) “Stade IV” posterior waving and multiple peaks with extreme stomal swelling.

From the third to the sixth hour, a hyper-reflectivity of the epithelial layer was maintained with the formation of a binary pattern characterized by two hyper-reflective lines (i.e., the ocular surface and the basal membrane) within a hypo-reflective layer. In all cases, there was an increase in stromal thickness with the appearance of hyper-reflective spots, and in 65.2% of cases (15/23) with an initial differentiation between the anterior (hyper-reflective) and posterior (hypo-reflective) stroma. An initial posterior waving was visible in 78.2% of cases (18/23) (Nioi-Napoli sign I° Stade- for more details see Figure 5). In 56.5% (13/23) of cases, several SS were detected at the stromal level. In 60.8% (14/23) of cases, the endothelium appeared hyper-reflective with the presence of multiple spots.

#### 3.3.2. The 6–9 h Interval

From the third to the sixth hour, the anterior surface of the cornea appeared as a single hyper-reflective layer in approximately 50% of cases. In all cases, there was a thickening of the stroma with the appearance of hyper-reflective spots in the context of the tissue. In 56.5% (13/23) of eyes, it was possible to identify the differentiation between posterior (hypo-reflective) and anterior (hyper-reflective) stroma. In 5/23 (21.7%) of cases, it was possible to identify a wave appearance on the posterior surface of the cornea with protruded zones into the anterior chamber (Nioi-Napoli II° Stade). Of interest, the endothelium was hyper-reflective with the presence of more intense spots in 100% of cases.

#### 3.3.3. Interval 9–12 h

From the 9th to the 12th hour, at the epithelial level, the phenomenon of the binary pattern was no longer observed. The anterior surface of the cornea appeared as a single hyper-reflective layer in 91.3% of all cases (21/23). In two cases, initial flaking of the epithelial cells was observed. In all cases, there was a thickening of the stroma with the formation of hyperreflective spots in its context and anterior–posterior differentiation. In 60.8% (14/23) of the posterior stroma, one or two peaks protruded in the anterior chamber (Nioi-Napoli III° Stade). In all cases, a hyper-reflectivity of the endothelium concerning the posterior stroma was maintained.

#### 3.3.4. The 12–17 h Interval

In all cases, from the 12th to the 17th hour, the epithelia remained hyper-reflective. Of note, in 34.7% (8/23) of cases, the epithelium presented initial but observable flaking with partial exposure to the basement membrane. In all cases, the stroma showed notable growth with structural inhomogeneity, antero-posterior differentiation, and hyperreflective spots. Moreover, all cases revealed a high waves pattern of the cornea’s posterior surface (Nioi-Napoli IV° Stade). On the other hand, SS were still visible in 14 out of 23 cases (60.8%). Compared to the previous intervals, we started to detect a loss of sphericity of the tissue with an initial deformation in two cases out of twenty three (8.6%).

**Figure 6:**
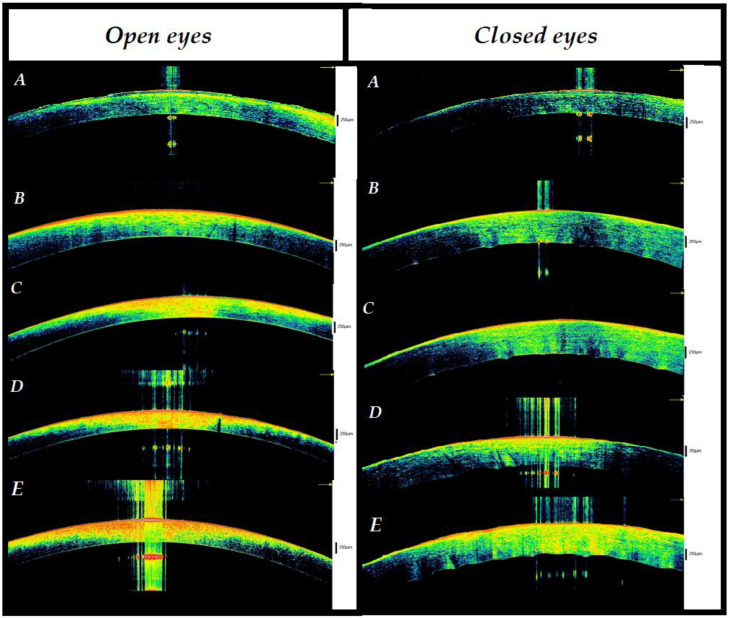
Comparison of changes over time between closed and open eyes. (**A**) Baseline. It includes the interval between death and the third hour without any change in the state of opening of the eyelids. The structure of the corneas appeared normal, similar to that of living people. Of interest, stromal striae (SS) are easily detected in a number of cases. (**B**) Interval between the third and sixth hour. The comparison showed the appearance of hyper-reflectivity in the anterior cornea in the open eye. At the level of the epithelium, the presence of the ‘binary sign’ was recorded in all cases. In closed eyes, on the other hand, a stromal thickening with initial waving of the posterior part of the cornea was observed. (**C**) In the open eyes there was, at the epithelial level, the presence of a single hyper-reflective layer that replaced the binary sign. The stroma was thinned and characterized by hyper-reflectivity in its anterior 3/4. In closed eyes, a thickening of the stroma with the presence of single peak that protruded posteriorly into the anterior chamber was recorded. (**D**) Interval between the ninth and twelfth hour. In the open eyes, further thinning of the stroma was observed with full thickness hyper-reflectivity in the central part. In closed eyes, a stromal thickening with differentiation between the anterior (hyper-reflective) and posterior (hyporeflective) portions was recorded and the presence of at least two peaks protruding in the posterior part was noted. (**E**) Interval between the twelfth and fifteenth hour. In open eyes, the initial presence of hyporeflectivity in the posterior stroma and the appearance of endothelial hyporeflectivity spots was observed. On the other hand, in closed eyes, a further important increase in stromal thickness was revealed with the presence of hyper-reflective spots in the tissue. Moreover, the presence of more protruding peaks in the posterior chamber was noted. With regard to the remaining signs, see Table 1 and Table 2 for details.

The main alterations affecting the closed eye are summarized in Table 2. A comparison between the morphological picture present in the different imports is proposed in Figure 6.

### 3.4. Influence of Environmental Conditions

The samples were tested in two seasons. Although the morgue temperature was kept at a constant level, it was inevitable to have a temperature differences between the experiments carried out in the summer season and those carried out in the winter season.

In particular, we observed that due to the greater exposure to dehydration, higher temperatures led to an early onset of ocular surface dehydration and, consequently, a more rapid thinning of the cornea in the eyes that were kept open. The drying phenomena had less of an impact on closed eyes.

**Figure 7:**
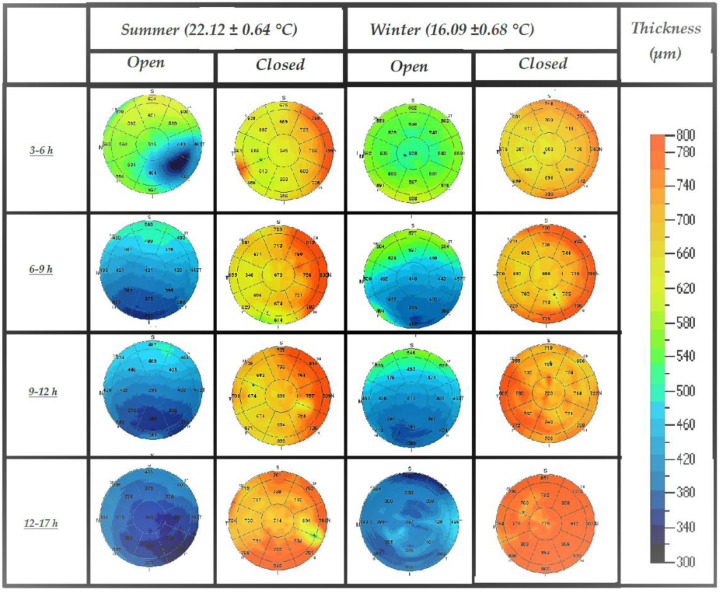
Pachymetric maps of central cornea (6 mm central area) at different postmortem intervals in closed and open eyes during the summer and the winter.

The pachymetric and morphological changes in closed eyes were instead more influenced by the presence of posterior edema probably linked to the hypoxic/anoxic physiological state that characterizes each body site in the post-mortem. From the morphological point of view, this condition manifested itself through a progressive thickening of the stroma (Figure 7).

## 4. Discussion

The corneal tissue in living people has fundamental characteristics, such as transparency (thus allowing the passage of light), a structure capable of maintaining a dioptric power, and a resistance that prevents foreign or pathogenic bodies from reaching the innermost ocular structures.

To maintain transparency in its central part, the cornea is not crossed by vessels, and nutrients arrive essentially through the vessels that reach the limbus and by a direct passage from the aqueous humor and tear film [21,22,23].

In living people, when the eyes are open, the oxygen that is dissolved in the tear film leads to oxygenation of anterior part of the cornea. Conversely, when the eyes are closed, the oxygenation of the cornea is maintained by the limbal vessels and the aqueous humor [24,25,26,27].

Clearly, the phenomenon of death involves a progressive alteration in the physiology of every tissue in the body, including the cornea. These phenomena are at first of an immediate abiotic type (arrest of circulation, arrest of breathing, and arrest of consciousness). Subsequently, consecutive abiotic types (dehydration, cooling, hypostasis, corpse rigidity, tissue acidification) occur. Finally, with time, transformative type phenomena begin to be seen [28,29].

To best understand post-mortal phenomena, it is important to acknowledge that macroscopic death does not simultaneously affect all the organism’s cells. The single cells that remain alive tend to oppose the changes that occur at the macroscopic level. However, theoretically, it is not possible to exclude that even in vertebrates there may be a sort of post-mortal cellular communication of a biochemical type through which the cells are progressively informed of the death of the macroscopic body (i.e., the end of the life of entire multicellular organism). This phenomenon has been described for example by a famous article on Caenorhabditis elegans, a nematode in which the existence of a cascade of cell death involving the calpain–cathepsin necrosis pathway that can drive organismal death was demonstrated. This message of macroscopic death was recorded in the aforementioned article with fluorimetric techniques that showed a burst of intense blue fluorescence (named “blue wave”), generated within intestinal cells by the necrotic cell death pathway [30].

The death, at the level of the cornea, involved the appearance of the immediate abiotic phenomena that are responsible for important changes in metabolism, oxygenation, and oxygen exchange.

From a metabolic point of view, the absence of nutrients from the blood and vitreous associated with hypoxia leads to alterations in the corneal tissues and the level of the aqueous and vitreous humor [31,32,33].

Hypoxia constitutes one of the fundamental factors regarding post-fatal alterations. Its main effect is to alter the acidity of the tissue, causing the failure of the ion transport channels that physiologically maintain homeostasis in the tissue. From the available studies addressing the effects of contact lenses, we know that the main effect of hypoxia is edema: in fact, the tissue (or rather the stroma) subjected to anaerobic metabolism tends to swell [34,35,36,37].

It is implied in the current study, by analyzing corpses rather than living beings, that the postmortem hypoxic/anoxic phenomena (which are omnipresent after death, affecting every part of the body) are also present in the cornea.

Another determining postmortem factor is dehydration. Without tears and blinking, the corneal surface is exposed to air [38].

### 4.1. Open Eye

Open eye status overexposes corneal tissue to dehydration. Tear production and tear film distribution on the ocular surface are lacking. Despite this, in the first 2–3 h, the presence of a residual film allows homeostatic maintenance of the physiological corneal morphology (“homeostatic balance” phase).

From the third hour, something changes. Mainly affecting the epithelium, a phase of cellular decompensation and progressive dehydration is evident with the progressive disappearance of the binary sign (“initial dehydration” phase).

From the 6th to the 9th hour, dehydration becomes evident, thus involving several corneal changes. First, in most cases, the epithelium with a single-track morphology appears as a single hyperreflective layer. The phenomenon of dehydration affects the anterior 3/4 of the stroma. The particular spherical conformation of the cornea and the physiological difference between the anterior and posterior stroma highlight the following two particular morphological signs: the hyporeflective linear- or alphabetic-shape SS and the sawtooth sign (Figure 3 and Figure 4). The first is the disclosure of an ultrastructural feature of the cornea, which becomes more easily detectable when the tissue is subjected to extreme dehydration [39,40,41]. The second is provided by the irregular expansion of dehydration favored by the spherical shape of the cornea and by the structural differences between the anterior and posterior stroma (“intermediate dehydration” phase).

In the interval between the ninth and the twelfth hour, dehydration reaches its peak with the hyper-reflective zone that arrives, in the central portion of the tissue, from the epithelium to the endothelium, with an accentuation of the phenomena described above (“advanced dehydration” phase).

From the twelfth hour, a new phenomenon appears: the posterior part of the stroma begins to become hypo-reflective. This phenomenon is probably due to the decompensation of the endothelium, which allows the passage of aqueous humor through the corneal tissue by diffusion. This results in an altered distribution of the fibers and macroscopically translates into an initial loss of tissue transparency (“endothelial decompensation” phase).

### 4.2. Closed Eye

During the interval between death and the third hour, as occurs in the open eye, and due to homeostasis systems such as endothelial pumps, there were no significant changes in the corneal tissue except for slow tear film evaporation (“homeostatic balance” phase) [42].

In the interval from the third to the sixth hour, the main alteration was the thickening of the stroma with differentiation between a hyper/hypo-reflective anterior part and a hypo-reflective posterior part. This is attributable to the passage of aqueous humor from the anterior chamber to the corneal tissue due to initial endothelial decompensation. This phenomenon also affects the posterior stroma and endothelium, which begin to assume a wavy morphology (Nioi-Napoli I°). The epithelium in some sections (probably due to the hypoxic suffering of the cells of the central layers of the epithelium) assumes a binary morphology with hyperreflective external and internal parts and hypo-reflective central parts. In more than half of the cases, the linear, X and V-shaped SS can be observed (“initial swelling” phase).

From the sixth to the ninth hour, in 50% of cases, the epithelium is represented by a single hyperreflective layer. There is also a further swelling of the stroma always due to endothelial decompensation with the appearance of at least one peak that protrudes into the posterior chamber. This appearance is also due to the structural difference between the anterior and posterior stroma, accentuating the gradual passage of aqueous humor from the anterior chamber [43,44,45]. The endothelium was hyperreflective with the presence of some spots in its context (“intermediate swelling” phase).

From the ninth to the twelfth hour, the characteristics of the epithelium remain unchanged. On the contrary, there continues to be a passage of aqueous humor to the tissue from the posterior chamber. The distinction between anterior hyper-reflective and posterior hypo-reflective stroma appears clearer. The differences in the arrangement of the stromal fibers mean that at least two peaks protrude into the anterior chamber in at least 60.8% of cases. However, sporadic hyperreflective spots are observed in the context of the stroma. The endothelium always appears hyper-reflective (“advanced swelling” phase).

From the twelfth hour, the epithelium remains unchanged while there is a significant increase in corneal thickening that can exceed 1000 µm. In some cases, this variation involves the loss of the physiological sphericity, while on the back, there are various peaks that assume a greater height than those described previously (Nioi-Napoli III-IV). It would seem that this process is provided by the definitive decompensation of the endothelium (which seems to remain hyper-reflective) with passage by direct diffusion of aqueous from the anterior chamber to the tissue. In the stromal context, there are hyper-reflective spots while the fibrillar lines take on disordered trends that are incompatible with maintaining physiological transparency (phase of endothelial failure or fibrillar disruption).

### 4.3. Influence of Environmental Conditions: Temperature and Humidity

Dehydration is one factor that we hypothesized would influence postmortem changes; it is evident that temperature and humidity play an important role, especially when the eye is open and fully exposed to the air. However, the scans of the study were minimally affected by this factor as they were performed at controlled temperatures (22.12 ± 0.64 °C in summer and 16.09 ± 0.68 °C in winter). Despite this, comparing the pachymetric maps shows slight anticipation of the phenomena in the open eyes when the scan occurs at a higher temperature. On the contrary, the phenomenon appears almost ineffective when OCT scanning is carried out on the eye when closed, and probably the hypoxic/anoxic postmortem status is the main factor that induces the changes.

### 4.4. Future Research and Practice Perspectives

The current study could have significant implications in the forensic and ophthalmological fields.

As far as forensics is concerned, this study represents the first work that morphologically described the process that leads to corneal opacification. In the future, more in-depth knowledge of the phenomenon could affect estimations of the postmortem interval (PMI) and some particular aspects of the crime scene (movement of the corpse involving opening or closing of the eyelids).

Additionally, from an ophthalmological point of view, the study offers important insights and represents a model for the effects of hypoxia. From a practical point of view, the results provide important information that is helpful in conserving the tissue in situ before explant. The data of the current study suggest that the lid closure status has little impact on OCT corneal morphology during the initial endothelial decompensation. After the third hour, on the contrary, the dehydration phenomena become prominent, thus favoring the morphological alteration of the tissue. The latter finding supports the current clinical practice of eye closure in cadavers in which a corneal transplant is expected (which is also a common practice of “pietas” toward deceased persons). Unfortunately, this study design did not include pharmacological interventions aimed at preserving the tissue longer in conditions of open or closed eye. The reversibility of the various postmortal alterations due to the different eyelid status was not evaluated. Clearly, regarding both the forensic and ophthalmological fields, further studies with larger sample sizes are now needed.

### 4.5. Limits of the Study

The current study has several limitations. First, because of ethical reasons, we could not study the corpses from the precise moment of death, and, as mentioned in the methods, the state of closure of the eyelids in the interval from 0 to 1.5 h was random. Second, the number of observations should also be extended to individuals with ocular and systemic pathologies to verify their influence on the phenomena described. Third, carrying out the study at a monitored temperature and within a certain range did not allow us to assess the effects of very high or very low temperatures, humidity, and ventilation on the manifestation of the alterations.

## 5. Conclusions

The postmortem study of the cornea by portable OCT allowed us to better understand the phenomena that lead to morphological–structural alterations of the tissue after death. However, further studies are necessary to apply the method for thanato-chrono-diagnosis in forensic field, and tissue conservation purposes in transplantation.

## Figures and Tables

**Table 1:** Main changes in the open eyes in interval postmortem from 0 to 17 h.

	***Epithelium***	***Stroma***	***Endothelium***	***Other Signs***
***0–3***	Presence of lacrimal tear	Physiological thickness.	Preserved	Nothing.
***3–6***	Lacrimal tear progressively disappears. Hyperreflectivity with a “binary” pattern.	Initial thinning with differentiation between the anterior (hyper-reflective) and posterior (hypo-reflective) parts.	Hyper-reflective whit spots.	Initial sawtooth and stromal striae are detectable in the stroma.
***6–9***	Single, hyperreflective layer.	Decrease in stromal thickness. Hyperreflectivity in ¾ of anterior tissue in 68.5% of cases.	Unchanged.	Unchanged
***9–12***	Unchanged	Hyperreflectivity reaches the endothelium and takes a trapezoid form.	Enhancing of hyperreflectivity.	Stromal striae (65.8%) Sawtooth sign (78,2%) Initial loosing of the spheric form of the tissue in 21.7% of cases
***12–17***	Unchanged	Rising of posterior stroma hypo-reflectivity that in some cases reaches the middle of the tissue.	Are evident areas of hypo-reflectivity.	Stromal striae (100%) Sawtooth sign (100%) Initial loosing of the spheric form of the tissue in 34.7% of cases

**Table 2:** Main changes in the closed eyes in interval postmortem from 0 to 17 h.

	***Epithelium***	***Stroma***	***Endothelium***	***Other Signs***
***0–3***	Presence of lacrimal tear	Physiological thickness.	Preserved	Nothing
***3–6***	Lacrimal tear disappears. Binary sign with hyperreflectivity of the outer and inner layer and hyperreflectivity in the middle.	Posterior waves (Nioi Napoli sign grade 0). Thickening of the stroma with initial differentiation between posterior and anterior tissue. In 69,5%, the posterior stroma assumes a wavy morphology.	Hyperreflective respect to the posterior stroma. Presence of Endothelial spots	V-shapes in 56.5% of cases. Nioi–Napoli I (21.7%)
***6–9 ***	Appears like a single, hyperreflective layer in 50% of cases. In 50% ‘binary morphology’.	Differentiation between anterior hyperreflective and posterior hypo-reflective stroma in 56.5% of cases. Presence of one tissue peak that protrudes in the anterior chamber (Nioi-Napoli 1)	Hyper-reflectivity with the presence of multiple spots.	Nioi–Napoli II (21.7%). Initial loosing of the spheric form of the tissue.in 34.7% of cases
***9–12***	Single hyperreflective layer in 91.3% of cases. In the 8.7% initial flaking of the epithelium.	Thickening of the stoma. Anterior-posterior differentiation in 100% of cases. Waving with one of two protruding peaks in 14 cases (60.8%).	Unchanged	Nioi–Napoli III (60.8%)
***12*** ***–*** ***17 ***	Single hyperreflective layer in 100%. Epithelium flacking in 34.7% of cases.	Important thickening, structural inhomogeneity with anterior-posterior differentiation and multiple hyporeflective spots. Posterior waving with more than two peaks in 100% of cases.	Hyperreflective.	Nioi–Napoli IV in 100% of cases. Stromal Striae in 60.8% of cases.

## Data Availability

The data presented in this study are available on request from the corresponding author (MN).
